# Diagnostic Utility of Copeptin in Pediatric Patients with Polyuria-Polydipsia Syndrome: A Systematic Review and Meta-Analysis

**DOI:** 10.3390/ijms251910743

**Published:** 2024-10-05

**Authors:** Diana-Andreea Ciortea, Carmen Loredana Petrea (Cliveți), Laura Bujoreanu Bezman, Iolanda Cristina Vivisenco, Sorin Ion Berbece, Gabriela Gurău, Mădălina Nicoleta Matei, Aurel Nechita

**Affiliations:** 1 Faculty of Medicine and Pharmacy, University “Dunarea de Jos” of Galati, 800201 Galati, Romania; diana.ciortea@ugal.ro (D.-A.C.); laura.bezman@ugal.ro (L.B.B.);; 2 Emergency Clinical Hospital for Children “Maria Sklodowska Curie”, 041451 Bucharest, Romania; 3 Emergency Clinical Hospital for Children “Sf Ioan”, 800487 Galati, Romania; 4 Faculty of Medicine, “Carol Davila” University of Medicine and Pharmacy, 030167 Bucharest, Romania; cristina.vivisenco@umfcd.ro; 5 Emergency Clinical Hospital for Children “Grigore Alexandrescu”, Department of Pediatrics, 011743 Bucharest, Romania

**Keywords:** copeptin, polyuria-polydipsia syndrome, pediatrics, diagnostic accuracy

## Abstract

Pediatric patients with polyuria polydipsia syndrome (PPS) represent a diagnostic challenge for clinicians because of the technical difficulties in performing the gold standard water deprivation test (WDT). Copeptin, a stable biomarker representing the C-terminal portion of the polypeptide chain of the antidiuretic hormone, is a reliable diagnostic tool. To assess the diagnostic accuracy of baseline copeptin dosing, arginine/hypertonic saline copeptin stimulation tests, and WDT. This study aimed to establish the diagnostic utility of copeptin in pediatric patients by distinguishing between central diabetes insipidus, nephrogenic diabetes insipidus, and primary polydipsia. Comparative and non-comparative primary studies published between January 2018 and August 2024 focusing on children were searched and included in PubMed, Cochrane Library, Web of Science, ScienceDirect, Scopus, and Google Scholar. The QUADAS-2 tool was used to assess the risk of bias and applicability. Meta-analyses used fixed effects models because of low heterogeneity and the HSROC model. Eleven studies were included with an overall low bias and no significant applicability concerns. The mean pooled sensitivity = 0.98 (95% CI: 0.936–1.025), pooled specificity = 0.947 (95% CI: 0.920–0.973), and AUC = 0.972 (95% CI: 0.952–0.992), indicating excellent diagnostic accuracy. Stimulation methods for copeptin dosing represent an effective and less invasive diagnostic test for children with PPS, and future development of standard copeptin testing protocols is needed.

## 1. Introduction

### 1.1. Background

Polyuria-polydipsia syndrome presents a diagnostic challenge, especially in the pediatric population. Currently, the gold standard for diagnosis is the water deprivation test, followed by administration of a synthetic antidiuretic hormone (ADH) molecule to evaluate the renal response to arginine vasopressin (AVP) secretion. This is typically assessed by monitoring urinary concentrations and serum osmolality elevation as indicators of a normal physiological response to dehydration [1]. It is well known that the results of this clinical test are subject to important technical limitations due to the potential discomfort it causes in young children, their noncompliance with all the steps included within this test, and the potential overlapping results. Therefore, it is sometimes difficult to establish the exact type and etiology of polyuria-polydipsia syndrome, especially when a more defined differentiation between partial central diabetes insipidus (CDI), nephrogenic diabetes insipidus (NDI), or primary polydipsia (PP) is needed.

Recently, the potential of copeptin dosing as an alternative diagnostic method has been demonstrated in clinical practice. Copeptin, synthesized in equimolecular proportion with ADH, represents a more reliable and stable biomarker for hypothalamic and renal function. Copeptin is the C-terminal part of the AVP precursor, pre-provasopressin, and consists of 164 amino acids. This precursor is synthesized by magnocellular and parvocellular neurons in the supraoptic and paraventricular nuclei of the hypothalamus, transported to the posterior pituitary gland, and released into the bloodstream under the influence of serum osmolality, which stimulates hypothalamic osmoreceptors [1,2]. The biochemical mechanism of its production involves cleavage into three parts: AVP, neurophysin II, and copeptin. AVP, also known as antidiuretic hormone (ADH), is a critical regulator of water homeostasis and blood pressure, producing these effects through three types of AVP receptors: V1a, V1b (or V3) and V2, which are heptahelical G protein-coupled receptors with different expression levels on the cell membrane in different tissues [2]. AVP is unstable and difficult to measure directly because of its short half-life and rapid degradation, with an estimated half-life of 15 min in serum samples. On the other hand, copeptin is more stable in circulation, making it a reliable surrogate marker for AVP secretion [3,4]. Clinical studies have shown that copeptin measurements can significantly improve the diagnostic rate of polyuria-polydipsia syndrome in pediatric patients. Differential diagnosis between CDI, NDI, and PP is essential for appropriate management of pediatric cases of polyuria-polydipsia syndrome. However, copeptin has not yet been definitively established as a single standard diagnostic test [4,5]. The diagnostic accuracy of copeptin in differentiating CDI, NDI, and PP has been validated in various studies, demonstrating its high sensitivity and specificity. For instance, Fenske et al. reported that stimulated copeptin levels had a sensitivity of 0.93 and a specificity of 0.96 in differentiating between PP and CDI. Additionally, copeptin levels are particularly useful in diagnosing NDI without requiring additional tests, such as the water deprivation test or hypertonic saline infusion [5,6]. This is significant for clinical practice as it simplifies the diagnostic process and reduces the patient burden.

Any treatment and management recommendations should be based on the best diagnostic methods, adapted for pediatric patients. Therefore, we systematically reviewed the most accurate, sensitive, and specific methods used for diagnostic tests in polyuria-polydipsia syndrome in the pediatric population. This review evaluates the diagnostic accuracy of copeptin dosing after hypertonic saline infusion or arginine stimulation, aiming to improve clinical practice and ease the diagnostic process for children.

### 1.2. Objectives 

The primary objective of this systematic review and meta-analysis was to assess the diagnostic accuracy and utility of baseline copeptin dosing, copeptin testing after hypertonic saline infusion, and arginine stimulation tests in pediatric patients presenting with polyuria-polydipsia syndrome and related symptoms. To achieve these objectives, this review was structured using the PIT framework.

**Population**: Children, infants, and adolescents (age < 18 years) presenting with polyuria-polydipsia syndrome, nocturia, and other symptoms of diabetes insipidus.

**Index Test**: Baseline dosing of copeptin, diagnostic test after hypertonic saline infusion or arginine stimulation of copeptin, and water deprivation test followed by arginine desmopressin administration.

**Target Condition**: Differential diagnosis of central diabetes insipidus (CDI), nephrogenic diabetes insipidus (NDI), primary polydipsia (PP), and other causes of polyuria-polydipsia.

We also designed a series of **secondary objectives** to provide complete evidence based on the diagnostic accuracy of copeptin and the water deprivation test and its contribution towards clinical decision-making and outcomes in pediatric endocrinology. 

We compared the diagnostic performance of copeptin dosing with that of the water deprivation test or other diagnostic methods for polyuria-polydipsia syndrome (PPS) patients. This was assessed by calculating the sensitivity and specificity of copeptin levels measured after hypertonic saline infusion, arginine stimulation, or other stimulation methods for copeptin dosing in the differential diagnosis of CDI, NDI, and PP. We compared this with the water deprivation test followed by arginine desmopressin administration or other traditional diagnostic methods.

We assessed the clinical utility of copeptin as a diagnostic biomarker by testing the feasibility and reliability of copeptin measurement, as it is less invasive and more child-friendly than the water deprivation test. We also tested the potential of copeptin levels for rapid and accurate differentiation between CDI, NDI, and PP, or other causes of polyuria in a clinical setting.

Finally, we identified gaps in existing studies by reviewing the current scientific literature and proposing future research directions to improve diagnostic protocols for pediatric patients with polyuria-polydipsia syndrome.

## 2. Methods

This review was registered in **PROSPERO International prospective register of systematic reviews** (ID CRD42024576715). The protocol was designed according to the Cochrane Handbook for Systematic Reviews of Diagnostic Test Accuracy [7]. The reporting was guided by the improved preferred reporting items for systematic review and meta-analysis adapted to the systematic reviews of diagnostic test accuracy PRISMA-DTA statement [8,9] (protocol available through PROSPERO registration https://www.crd.york.ac.uk/PROSPEROFILES/576715_PROTOCOL_20240822.pdf, accessed on 2 August 2024).

### 2.1. Eligibility Criteria

#### 2.1.1. Study Characteristics

Population

We included studies performed with children, infants, and adolescents with the following clinical symptoms: polyuria, polydipsia, nocturia, electrolytic imbalances, and other related symptoms of central diabetes insipidus, nephrogenic diabetes insipidus, or primary polydipsia.

The exclusion criteria were studies that exclusively referred to disorders not directly related to diabetes insipidus, as well as other definite causes of PPS, or studies assessing only adult patients. Moreover, patients with hyperosmolar polyuria, such as those with diabetes mellitus, were excluded to preserve the specificity of this review.

Index tests 

We considered the following index tests:

- Copeptin dosing: We selected studies that measure baseline copeptin levels and studies that assessed copeptin levels after hypertonic saline infusion or arginine stimulation. The protocol aims to achieve a target serum sodium level of approximately 150 mmol/L, corresponding to a serum osmolality of approximately 300 mOsm/kg [6,10].
- Water Deprivation Test: We considered all studies that utilized a standardized water deprivation protocol which involves depriving the patient of fluid for up to eight hours or until a 3% loss in body weight is achieved. During the test, plasma osmolality was measured at regular intervals to ensure adequate increase in endogenous vasopressin release. The urine volume and osmolality were monitored throughout the test. Following the fluid deprivation test, the patient was administered arginine desmopressin, a synthetic form of vasopressin, and urine volume and osmolality were measured to assess the response to exogenous vasopressin [1,5,6].
- Other relevant alternative diagnostic tests were conducted for patients presenting with diabetes insipidus-related symptoms.

Target condition: 

All patients were eligible if they presented with hippo-osmolar polyuria, associated with secondary polydipsia, due to inadequate secretion of ADH or an abnormal renal response or other related symptoms. Patients with hyperosmolar polyuria such as those with diabetes mellitus were excluded.

Reference standards:

- For copeptin dosing, studies had to include specific determination of baseline copeptin levels. Another assessed test was the copeptin levels test after intravenous infusion of 3% NaCl solution, as well as the arginine stimulation test or other copeptin stimulation methods. These mechanisms aim to achieve a target serum sodium level of approximately 150 mmol/L, corresponding to a serum osmolality of approximately 300 mOsm/kg.
- Additionally, studies comparing copeptin with other traditional diagnostic methods such as the water deprivation test were considered. This test is usually extended for 8 h and is followed by arginine desmopressin administration in order to assess the urinary response or ADH. To be included, the study should have recorded serum sodium levels ranging from 145 mmol/L to 150 mmol/L and urinary osmolality ranging from 300 to 1200 mOsm/kg.

These reference standards had to be included in the testing protocols because they are considered optimal for stimulating hypothalamic osmoreceptors leading to ADH release and subsequent renal urinary concentration as a physiological response.

Study design: 

Comparative Primary Study Designs: Mainly, we considered including comparative primary study designs in which all patients undergo all tests or patients are randomized to different tests.

Non-comparative Primary Studies: Due to the limited number of eligible studies, the decision was made to extend the eligibility criteria to include other types of studies. Non-comparative primary studies in which only one index tests were investigated were also included. These studies were selected based on similar populations, diagnostic pathways, and reference standards to minimize bias in our comparative DTA review. 

Other studies included: Due to the reduced number of such studies, especially in the pediatric population, we also considered randomized controlled trials (RCTs), cohort studies, case–control studies, cross-sectional studies, observational studies, and case series.

#### 2.1.2. Report Characteristics

The review was restricted to studies published in the last 6 years onward (between January 2018 and August 2024) because copeptin has been widely used for the systematic diagnosis of various types of polyuria-polydipsia syndrome in children since 2018. There were no restrictions on the language of publication. Unpublished manuscripts were not included. However, conference abstracts reporting relevant statistical data were eligible for inclusion to cover all relevant studies conducted on this subject. 

**Outcome Measures:** The primary outcome measures were the diagnostic accuracy measures, such as Sensitivity, Specificity, Positive Predictive Value (PPV), Negative Predictive Value (NPV), Likelihood Ratios, and Area Under the Curve (AUC). The accent was on the specificity and sensitivity of the main methods (the water deprivation test and copeptin baseline dosing, as well as IV hypertonic saline infusion or arginine stimulation tests) used for diagnostic of polyuria-polydipsia syndrome in pediatric population, adolescents, and young adults.

### 2.2. Information Sources 

This systematic review is based on a comprehensive and in-depth study of the available scientific literature. The review was conducted from March to August 2024, adhering to the recommendations of the Preferred Reporting Items for Systematic Reviews and Meta-analyses (PRISMA 2020) statement, modified to reflect the requirements for reporting diagnostic test accuracy studies in systematic reviews [9,11,12].

All the information was searched in databases accessed through the e-information platform.

The following electronic databases were searched for relevant studies: PubMed, covering biomedical literature from MEDLINE, life science journals, and online books; Cochrane Library and Web of Science, providing access to multiple databases that reference cross-disciplinary research, such as ScienceDirect Freedom Collection, Scopus, and Google Scholar.

### 2.3. Search Strategy

The search strategy utilized Medical Subject Headings (MeSH) terms and keywords related to polyuria-polydipsia syndrome and its diagnostic tests. The following terms were used, combined with Boolean operators (AND, OR) to ensure a comprehensive search: “Polyuria”, “Polydipsia”, “Nocturia”, “Diabetes insipidus”, “Diabetes insipidus nephrogenic”, “Arginine vasopressin”, “C-terminal provasopressin”, “Water deprivation test”, “Diamino arginine vasopressin”, “Copeptin”, “Diagnostic accuracy”.

This review follows the guidelines for describing search strings for systematic reviews in the form of Preferred Reporting Items for Systematic Review and Meta-Analysis Search (PRISMA-S) [13], ensuring that the search strategies are transparent and reproducible. Each search strategy was documented meticulously, including the database, search terms, date of the last search, and number of retrieved results. The searches were tailored to each database’s specific indexing system and capabilities. The final search was conducted on 3 August 2024. Articles not directly related to the main diagnostic tests for polyuria-polydipsia syndrome in children were excluded based on their relevance to the review. No unpublished or ongoing studies were included, and no other grey literature was considered for this review. All MeSH keywords and search strategies were adapted according to the specifics of each database, as represented in Appendix A—Table A1 (systematic review and meta-analysis search strategy are available through PROSPERO https://www.crd.york.ac.uk/PROSPEROFILES/576715_STRATEGY_20240822.pdf, accessed on 2 August 2024).

### 2.4. Study Selection Metodology

Initial screening of search results based on titles and abstracts was independently performed by two reviewers (D.-A.C. and L.C.P.). All eligible studies were assessed using full-text articles, and decisions regarding inclusion were also independently made. We also manually checked the reference lists of all included studies for additional citations. The extracted data were compared, and any discrepancies were resolved through discussion. If a consensus could not be reached, a third reviewer (G.G.) was consulted. In the first step, we selected 126 studies that were filtered down to 26 studies. After full-text assessment, only 11 studies were included in the meta-analysis based on their relevance to our main objective and completeness of reported data. 

### 2.5. Data Collection Process

Before full data extraction, the extraction form was piloted in two studies that were not included in this review to ensure clarity and consistency in data collection. 

The data extraction form was extracted in duplicate and included the following key elements:

**Study Identification**: Author(s), year of publication, journal.

**Participant Characteristics**: Age, clinical presentation, and inclusion/exclusion criteria.

**Index Test Details**: Details of the copeptin determination that clearly defines the protocols, including hypertonic saline or arginine stimulation, and other reference tests.

**Reference Standards**: The applied criteria and methods used to ascertain the diagnosis, clearly described, with reference to polyuria, polydipsia, serum sodium concentrations, or urinary osmolality.

**Diagnostic Accuracy Measures**: Sensitivity, Specificity, Positive Predictive Value (PPV), Negative Predictive Value (NPV), Likelihood Ratios, and Area Under the Receiver Operating Characteristic Curve (AUC).

**Results**: Individual data (raw data) are presented in detail in 2 × 2 tables with true positives, false positives, true negatives, false negatives, summary statistics, and all subgroup analyses.

**Quality Assessment**: The QUADAS-2 tool was used to assess the risk of bias and applicability of each study.

### 2.6. Definitions for Data Extraction 

All definitions used for the data extraction process were meticulously revised to ensure that we captured comprehensive and relevant information from each study. This approach allowed for a robust comparison of the diagnostic accuracy of copeptin versus traditional methods in the diagnosis of pediatric polyuria-polydipsia syndrome. 

To diagnose CDI, we considered a baseline copeptin value of <2.6 pmol/L [3,10,14]. Additionally, a low plasma copeptin level of <4.9 pmol/L obtained as a result of hypertonic saline infusion indicates central DI, both partial and complete forms. A higher level of ≥4.9 pmol/L indicates primary polydipsia [3,14]. Some studies reported a cut-off value of 6.5 pmol/L for hypertonic saline-induced copeptin levels because of its higher diagnostic accuracy, which we also took into account as equivalent [3,6]. Furthermore, a very high baseline copeptin value of >21.4 pmol/L is indicative of nephrogenic diabetes insipidus (NDI), encompassing both partial and complete forms [1,3,14].

### 2.7. Risk of Bias and Applicability 

The QUADAS-2 (Quality Assessment of Diagnostic Accuracy Studies-2) tool was used to assess the risk of bias and applicability concerns in individual studies. Two reviewers independently evaluated each study and discrepancies were resolved by a third reviewer. Detailed methods regarding patient selection, index test, reference standards, and main observations are provided in Appendix B.

### 2.8. Principal Diagnostic Accuracy Measures

In this systematic review, we assessed the following principal diagnostic accuracy measures for evaluating the performance of copeptin as a diagnostic test in pediatric patients with polyuria-polydipsia syndrome: (1) **Sensitivity** to measure the proportion of actual positives, patients with PPS correctly identified by the test (copeptin) to have the actual condition such as CDI; (2) **Specificity** for the proportion of actual negatives, patients correctly identified by the test (copeptin) to not have the condition such as ruling out the NDI or PP; (3) **Positive Predictive Value (PPV):** representing the proportion of positive test results for copeptin that are true positives, meaning that the test correctly identifies those out of all positive results given; (4) **Negative Predictive Value (NPV)** for the proportion of negative test results that are true negatives, meaning the copeptin test correctly identifies patients who do not have the condition out of the negative results given by the test; (5) **Likelihood Ratios (LR)** with LR+ (positive Likelihood Ratio) and LR- (negative Likelihood Ratio) indicating the test’s ability to correctly identify the condition from the patients with positive or negative tests; and (6) **Area Under the Receiver Operating Characteristic Curve (AUC)** for measuring the test’s overall ability to discriminate between those with and without the condition.

### 2.9. Data Handling for Synthesis of Results 

We used several methods to handle data, combined results from various studies, and described the variability between studies in this systematic review.

- **Standardization of Target Conditions:** Definitions of conditions such as CDI, NDI, and PP were standardized using clinical criteria across studies.
- **Harmonization of threshold**: Different thresholds for test positivity were harmonized, and subgroup analyses were performed according to different diagnostic methods. The use of Hierarchical Summary Receiver Operating Characteristic (HSROC) modeling further allowed us to account for and understand these threshold effects, providing a more nuanced analysis of diagnostic accuracy across studies.
- **Indeterminate results:** Clear definitions and sensitivity analyses were used for copeptin levels within a borderline range, and statistical adaptations were performed for undefined values when necessary.
- **Meta-Analysis techniques:** These were carried out using fixed effects models applied for the assessment of measures of diagnostic accuracy because the studies showed low heterogeneity. Heterogeneity was assessed using the I^2^ statistic, Cochran’s Q test, and Tau-squared (τ^2^) test. Statistical analyses were performed following standard meta-analysis protocols to ensure robust and reliable estimates using forest plots to visually compare diagnostic accuracy. Furthermore, we used the HSROC model to assess threshold variability and to obtain a deeper insight into diagnostic accuracy across studies [7].

### 2.10. Additional Analyses

In this systematic review and meta-analysis, we focused primarily on evaluating the overall diagnostic accuracy of copeptin in pediatric patients with PPS. To achieve this, we performed a detailed sensitivity analysis to account for the potential impact of risk of bias in the included studies. Another aspect of additional analysis was the handling of studies that included both pediatric and non-pediatric patients. Therefore, we conducted a sensitivity analysis excluding these studies to assess their influence on the overall results. Furthermore, we conducted subgroup analyses to evaluate the diagnostic accuracy of various methods used to diagnose PPS (e.g., baseline copeptin levels, copeptin after stimulation, and water deprivation tests). The Shapiro–Wilk test was used to evaluate the normality of the data, and because the data were not normally distributed due to smaller sample sizes in certain groups, the Kruskal–Wallis test, a non-parametric alternative to ANOVA, was employed. This test was used to compare sensitivity, specificity, positive predictive value (PPV), and other diagnostic metrics across subgroups, as a normal distribution could not be assumed. 

We conducted a data analysis using Python in the IDLE (Python 3.14) environment, which offers a reliable number of statistical capabilities. The key libraries used included NumPy and Pandas for data manipulation, SciPy for statistical analysis, and Matplotlib and Seaborn for data visualization. Advanced AI language models helped us in code generation to enhance precision and efficiency, with experts reviewing all processes to guarantee validity. To ensure the reliability of the results, key analyses were cross-checked using RStudio software (R version 4.4.1 (2024-06-14)) or IBM SPSS (version 29), confirming the consistency of the findings derived from Python scripts.

## 3. Results

### 3.1. Study Selection Results 

**Scheme 1:**
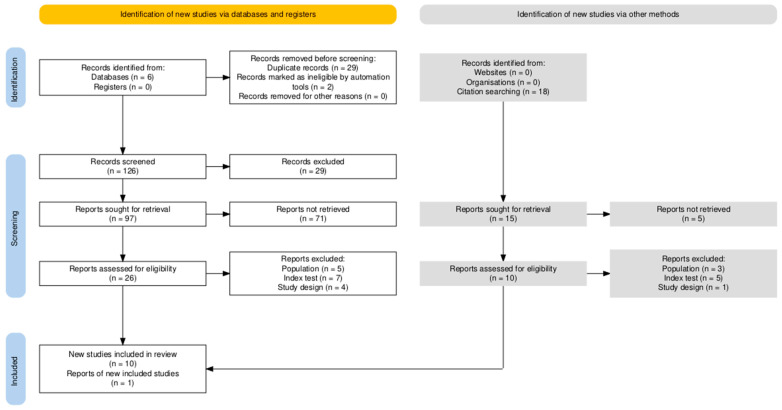
PRISMA-DTA flow diagram.

After database search, we identified a total of 126 records; after the removal of 29 duplicates, 97 records were screened and assessed for eligibility. As mentioned in the PRISMA flow diagram (Scheme 1) [15], 71 articles were initially excluded based on the title and abstract because they did not specifically focus on pediatric patients or adolescents and diagnostic methods for PPS. Further refinement was performed for the 26 articles selected for the full-text assessment. Several studies did not meet the index tests, by not using the specified diagnostic tests or biomarkers (Copeptin, Water Deprivation Test, Arginine Vasopressin, C-Terminal Provasopressin) or being literature reviews or case reports. Finally, 11 studies were included in our meta-analysis.

The main exclusion reasons were categorized according to PIT:

- Population:

Studies focusing on general DI management or treatment and patient perspective, rather than specific diagnostic methods.

Some studies that were not based on pediatric population with polyuria:

- Index Test:

Studies that do not address the diagnostic methods of our interest.

- Target condition:

Several studies that exclude CDI and NDI based on copeptin dosing; pathology was not of interest to us.

### 3.2. Study Characteristics

We identified 11 key studies based on relevance to the research question, being original research articles, focusing on the pediatric population, as well as completeness of data, sample size, clinical settings, and recent high-impact research for the analysis. We described them in detail to give an insight into the study design, population, and diagnostic methods (Table 1).

### 3.3. Quality Assessment and Publication Bias

#### QUADAS-2 Tool

It was used to assess the quality of the diagnostic accuracy studies. It evaluates the risk of bias and applicability concerns in four key domains. Detailed assessment is available in Appendix C.

For each of the four domains, we obtained the following results:

1. Patient Selection:

We identified a low risk of bias in most studies because the process of patient selection was clearly defined and was performed in a prospective way (Fenske et al., 2018, Tuli et al., 2023, Binder et al., 2023, Tuli et al., 2018, Nofal et al., 2023, Gippert et al., 2023) [10,17,19,21,23,24]. More than half of the studies were multi-centered, thus reducing the selection bias. A moderate risk of bias was identified in the studies conducted by Pedrosa et al., 2018, Kitamura et al. (2022), March et al. (2018), and Bonnet et al. (2024) [16,20,22,25], which were performed in a retrospective or single-center manner, which potentially limits the representativeness and may introduce some selection bias. Likewise, Bonnet et al. (2021) [25] performed a study in a single center with a moderate risk of bias.

2. Index Test:

A low risk of bias was obtained because index tests (standardization of copeptin measurement after arginine stimulation, hypertonic saline infusion, and water deprivation tests) were applied uniformly in all studies, with well-documented methodology. None of the studies showed bias in interpretation; hence, the risk of bias remained low in this domain.

3. Reference Standard:

A moderate risk of bias was obtained in most of the studies, because the reference standard for clinical diagnosis was based on expert consensus. Although this is a relatively robust approach, it could still be open to potential subjectivity that might rest on expert judgment. Very few studies obtained a low risk of bias, such as Fenske et al., 2018, Tuli et al., 2023, and Binder et al., 2023 [10,17,19], because the authors clearly provided diagnostic criteria and appeared to have applied the reference standard with a fair degree of blind conduct.

4. Timing and Flow:

Patient flow was clearly recorded in all studies, the appropriate interval between the index test and reference standard was maintained, there were minimal losses to follow-ups, and procedures were applied consistently. This makes it close to the low-risk bias domain.

**Figure 1:**
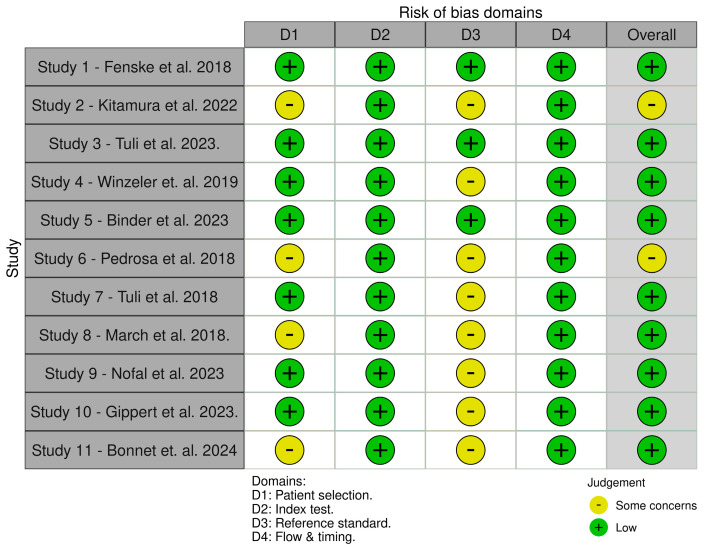
QUADAS-2 assessment—Study 1 [10], Study 2 [16], Study 3 [17], Study 4 [18], Study 5 [19], Study 6 [20], Study 7 [21], Study 8 [22], Study 9 [23], Study 10 [24], Study 11 [25].

Overall, all studies had a low to moderate risk of bias in all four domains of QUADAS-2 (Figure 1). The quality of the designs is supported by good standards of execution for index tests with clear documentation of the procedures, which strengthens the reliability of the findings. The main concerns arise from the domains of patient selection and reference standard. This is because most studies include single-center settings and retrospective designs, which might have further introduced bias into the consensus diagnosis by expert opinion.

### 3.4. Results of Individual Studies

For each analysis in all 11 studies, we extracted the unique combination of index tests, reference standards, and positivity thresholds, as well as the sensitivity and specificity that were provided, and adequately calculated the results for 2 × 2 data tables as seen in Table 2.

Further, for diagnostic accuracy estimates, we calculated and assessed Sensitivity, Specificity, Positive Predictive Value (PPV), Negative Predictive Value (NPV), Positive Likelihood Ratio (LR+), Negative Likelihood Ratio (LR−), and Area Under the Receiver Operating Characteristic Curve (AUC) for each study, as described in Table 2. Moreover, we provided confidence intervals (CIs) for all the diagnostic accuracy estimates. To obtain a general evaluation of the studies, an HSROC model was applied to account for variations in diagnostic thresholds across studies.

In the 11 studies included in our review and meta-analysis, we obtained high sensitivity and specificity ranging from 95% to 100% in the former and from 85% to 100% in the latter. The diagnostic accuracy for each index test was good to excellent in most of the studies, as confirmed by AUC values ranging from 0.93 to 1.00. Most of the studies showed very good diagnostic potential, especially Binder et al., 2023 [19], with the best performance, AUC = 1.00, followed by Tuli et al., 2018 [21] with an AUC of 0.99 and Winzeler et al., 2019 [18] with an AUC of 0.98. In general, the confidence intervals calculated for sensitivity and specificity were narrow, suggesting precise estimates. However, some studies (e.g., Kitamura et al., 2022 [16]) had wide intervals due to smaller sample sizes. Regarding Likelihood Ratios with values for LR+ > 7 and LR− ≅ 0, found in most of the studies suggest, overall, that these tests are good for ruling in and ruling out polyuria polydipsia syndrome, like CDI.

**Figure 2:**
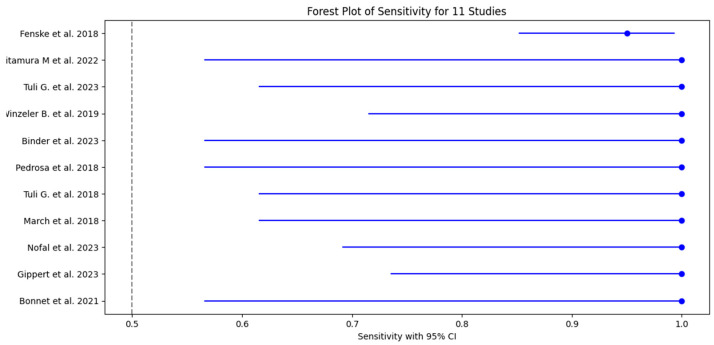
Forest plot of Sensitivity—illustrating the sensitivity of copeptin across 11 studies. The pooled sensitivity is 0.96, indicating high diagnostic accuracy in identifying true positive cases. (Fenske et al., 2018 [10], Kitamura et al., 2022 [16], Tuli et al., 2023 [17], and Winzeler et al., 2019 [18], Binder et al., 2023 [19], Pedrosa et al., 2018 [20], Tuli et al., 2018 [21], March et al., 2018 [22], Nofal et al., 2023 [23], Gippert et al., 2023 [24], Bonnet et al. [25]).

**Figure 3:**
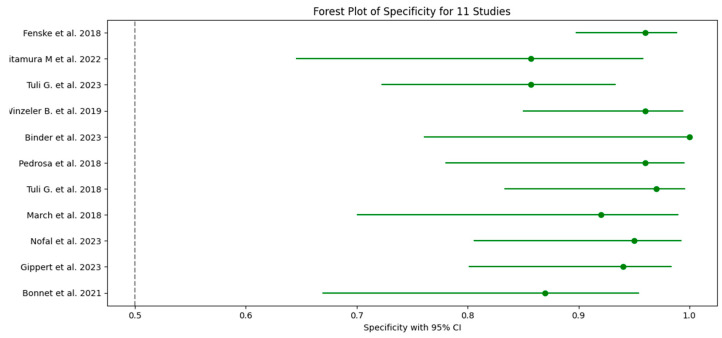
Forest plot of Specificity—showing the specificity of copeptin across 11 studies. The pooled specificity is 0.95, demonstrating strong diagnostic capability in correctly identifying true negative cases (Fenske et al., 2018 [10], Kitamura et al., 2022 [16], Tuli et al., 2023 [17], and Winzeler et al., 2019 [18], Binder et al., 2023 [19], Pedrosa et al., 2018 [20], Tuli et al., 2018 [21], March et al., 2018 [22], Nofal et al., 2023 [23], Gippert et al., 2023 [24], Bonnet et al. [25]).

To further illustrate the variability and precision of the sensitivity and specificity estimates across the studies, forest plots were generated (Figure 2 and Figure 3). These plots allowed for a direct comparison between studies regarding the diagnostic performance of copeptin in pediatric polyuria-polydipsia syndrome.

Forest plots for Sensitivity and Specificity provided a comprehensive overview of the diagnostic performance of copeptin within the selected studies, as shown in more detail in Figure 2 and Figure 3. For each study, Sensitivities, that is, true positive rates, and Specificities, which are the true negative rates, along with their corresponding 95% confidence intervals were reported. The plots allowed for a direct comparison between studies regarding the precision and variability of these estimates, focusing on individual measures.

**Figure 4:**
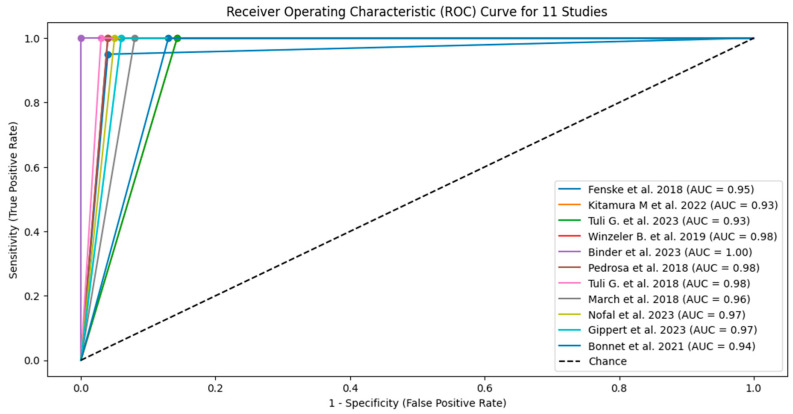
ROC plot for the selected studies (Fenske et al., 2018 [10], Kitamura et al., 2022 [16], Tuli et al., 2023 [17], and Winzeler et al., 2019 [18], Binder et al., 2023 [19], Pedrosa et al., 2018 [20], Tuli et al., 2018 [21], March et al., 2018 [22], Nofal et al., 2023 [23], Gippert et al., 2023 [24], Bonnet et al. [25]).

To further evaluate the diagnostic performance of copeptin across the studies, from a different perspective, we generated a Receiver Operating Characteristic plot (Figure 4). It graphically shows how the sensitivity varies with 1—Specificity. Each curve corresponds to a study and the Area Under the Curve is a summary measure of overall diagnostic accuracy. The higher the AUC, the better the diagnostic performance: an AUC of 1.0 represents a perfect test. The studies with curves closer to the upper-left corner showed a higher diagnostic accuracy and better Sensitivity with Specificity.

### 3.5. Test Accuracy and Variability

To assess the diagnostic accuracy of copeptin in pediatric patients with polyuria-polydipsia syndrome, 11 studies were analyzed based on the main diagnostic accuracy metrics.

The sensitivity ranged from 95% in Fenske et al., 2018 [10] to 100% in Kitamura et al., 2022 [16], Tuli et al., 2023 [17], and Winzeler et al., 2019 [18], which is a clear indication of an absolutely high ability of the copeptin test to detect true positives.

The specificity ranged from 85.7% with a 95% CI of 66.89–95.45% in Bonnet et al., 2021 [25], to 100% with a 95% CI of 76.03–100% in the Binder et al., 2023 [19], which underlines variability of test performance concerning true negatives.

Variability was shown by forest plots for Sensitivity and Specificity across studies with confidence intervals that express the precision of such estimates. Several studies such as Winzeler et al., 2019 [18] had high narrow confidence intervals, with a specificity of 95% CI: 84.98–99.46%, showing more homogeneous test performance. Other studies, such as that by Bonnet et al., 2021 [25], have had wide-ranging intervals. For example, specificity with 95% CI: 66.89–95.45% shows a high variability in its accuracy.

Values of AUCs that highlight the overall diagnostic performance ranged from 0.93 in Kitamura et al., 2022 [16], Bonnet et al. [25] and Tuli et al., 2023 [17] to 1.00 in Binder et al., 2023 [19]. Most studies have presented AUC values above 0.95, resulting in a collectively high overall diagnostic accuracy of copeptin in this patient population. The AUCs of the individual studies were given together with their corresponding 95% CI, stating the range of uncertainty of these estimates. For example, in the study by Fenske et al., 2018 [10], an AUC of 0.95 was reported, with a 95% CI of 0.88–0.98, indicating very good diagnostic performance.

To consider the variability between studies, especially their thresholds for test positivity, which sometimes the traditional meta-analysis may not fully capture, we also used HSROC analysis. This analysis provided a more comprehensive understanding of diagnostic accuracy by modeling the relationship between sensitivity and specificity across studies.

### 3.6. Meta-Analysis

Results

A meta-analysis of diagnostic performance across all studies was conducted, pooling the Sensitivity, Specificity, and AUC values across the studies. The variances of each assessed measure, as well as the weight for each study were precisely calculated using a fixed effects model (Table 3).

**Figure 5:**
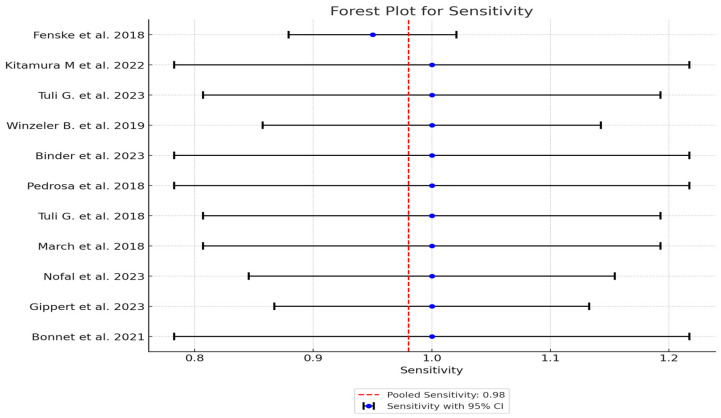
Forest plot of pooled sensitivity for all 11 studies. The red dashed line represents the overall pooled sensitivity estimate of 0.98 (95% CI: 0.936–1.025). The individual study estimate sensitivity values (blue dots) are shown with 95% confidence intervals represented as lines, highlighting the consistency of high sensitivity across most studies. (Fenske et al., 2018 [10], Kitamura et al., 2022 [16], Tuli et al., 2023 [17], and Winzeler et al., 2019 [18], Binder et al., 2023 [19], Pedrosa et al., 2018 [20], Tuli et al., 2018 [21], March et al., 2018 [22], Nofal et al., 2023 [23], Gippert et al., 2023 [24], Bonnet et al. [25]).

The pooled sensitivity had a high value of 0.98, proving that copeptin is highly effective in correctly identifying cases of polyuria-polydipsia syndrome, as shown in Figure 5. The forest plot illustrates consistency among the studies; high sensitivity values were observed in most of the studies, as indicated by the overlapping of their confidence intervals. However, some studies such as Kitamura et al., 2022 [16] have rather wide intervals. This can be attributed to the small sample size, which may have resulted in less precision of those estimates. Taken together, the evidence suggests a pooled sensitivity that provides very high probability that this diagnostic test for copeptin will correctly identify true positive cases of PPS.

**Figure 6:**
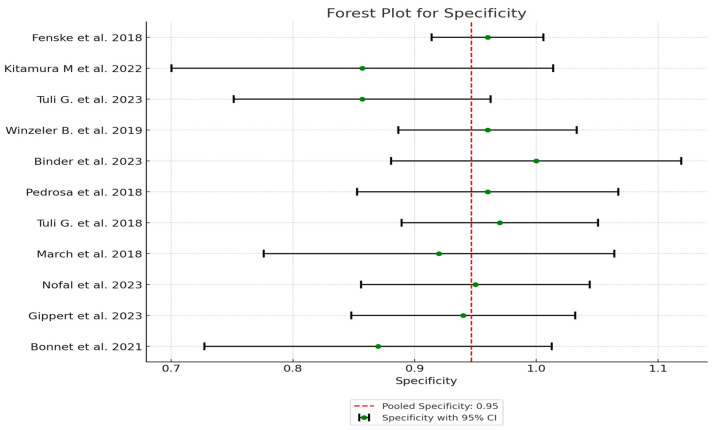
Forest plot of pooled specificity for 11 studies. The red dashed line represents the overall pooled specificity estimate of 0.95 (95% CI: 0.920–0.973). Each study’s specificity represented by a green dot is shown with its respective 95% confidence interval (represented by lines), demonstrating variability in test performance across studies, with most estimates clustering near the pooled estimate. (Fenske et al., 2018 [10], Kitamura et al., 2022 [16], Tuli et al., 2023 [17], and Winzeler et al., 2019 [18], Binder et al., 2023 [19], Pedrosa et al., 2018 [20], Tuli et al., 2018 [21], March et al., 2018 [22], Nofal et al., 2023 [23], Gippert et al., 2023 [24], Bonnet et al. [25]).

In parallel, the pooled specificity was 0.947, thus showing the strong capability of copeptin to correctly identify true negative cases, thereby minimizing the risk of a false positive diagnosis (Figure 6). The overall consistency observed in the pooled estimate indicates that copeptin is highly accurate in correctly identifying true negatives, thereby reducing the risk of false positive diagnoses.

**Figure 7:**
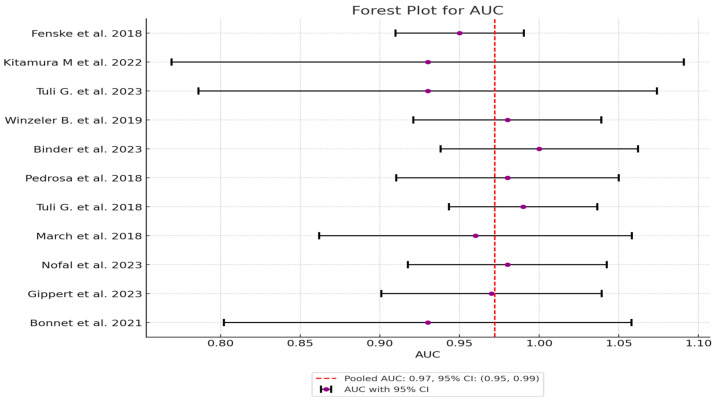
Forest plot of AUC (Area Under the Curve) across the 11 studies. The red dashed line represents the overall pooled AUC estimate of 0.97 (95% CI: 0.95–0.99), highlighting the high diagnostic accuracy of copeptin across studies. The tight clustering of AUC values (purple dots, associated with lines representing CI) around the pooled estimate further emphasizes the reliability and consistency of copeptin as a diagnostic tool for polyuria-polydipsia syndrome in pediatric patients. (Fenske et al., 2018 [10], Kitamura et al., 2022 [16], Tuli et al., 2023 [17], and Winzeler et al., 2019 [18], Binder et al., 2023 [19], Pedrosa et al., 2018 [20], Tuli et al., 2018 [21], March et al., 2018 [22], Nofal et al., 2023 [23], Gippert et al., 2023 [24], Bonnet et al. [25]).

These findings were confirmed by an AUC value of 0.972, underscoring very high diagnostic accuracy due to excellent discriminative power between cases and non-cases (Figure 7). The AUC values across the studies were closely clustered around the pooled estimate, indicating the consistent diagnostic performance of copeptin across different populations and study designs.

#### 3.6.1. HSROC Model Results

We performed another meta-analysis using a Hierarchical Summary Receiver Operating Characteristic (HSROC) model to evaluate the diagnostic accuracy of copeptin across the 11 studies and to obtain a more accurate image of copeptin efficiency that a traditional meta-analysis may not comprise. This model inherently incorporates random effects, which means that it accounts for the between-study variability. This aspect is particularly important in diagnostic accuracy assessment where different studies might use different thresholds for defining the positive diagnosis based on the copeptin level. In consequence, the HSROC model accounts for the variability in thresholds and provides a more nuanced summary of the diagnostic accuracy that reflects the variability between studies, even if we obtained a low overall heterogeneity. Using the HSROC model, we estimated a logit-transformed sensitivity of 2.7873 and a logit-transformed false positive rate of −2.4335. On the probability scale, this corresponded to an overall sensitivity of 0.942 and specificity of 0.92. These estimates suggest that while the traditional meta-analysis pooled sensitivity of 0.98 (95% CI: 0.936–1.025) and specificity of 0.947 (95% CI: 0.920–0.973) were slightly higher, the HSROC model helped us understand the diagnostic accuracy by modelling the relationship between sensitivity and specificity across studies by providing a Summary Receiver Operating Characteristic (SROC) curve.

**Figure 8:**
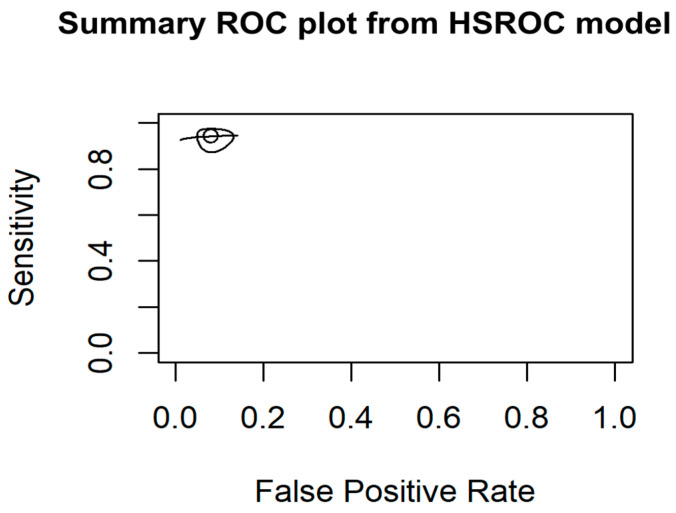
SROC plot from HSROC model—shows diagnostic performance of copeptin across all 11 studies. Circles represent summary estimate of sensitivity and specificity across studies. Lines around circle represent confidence regions for these estimates, showing uncertainty around summary points. *x*-axis (false positive rate) and *y*-axis (Sensitivity) are used to plot the trade-off between sensitivity and specificity.

The SROC curve obtained by the HSROC model (Figure 8) illustrates the relationship between sensitivity and false positive rate, indicating that copeptin generally demonstrates high diagnostic accuracy. However, the curve also suggests that there is some variability in specificity across studies, highlighting the importance of considering the context in which the test is applied. The SROC reflects the inherent trade-offs between these measures across different studies and thresholds, suggesting that while the overall diagnostic performance of copeptin is high, there is some variability in how sensitivity and specificity are balanced across different clinical settings.

#### 3.6.2. Assessment of Heterogeneity

For heterogeneity assessment, Cochran’s Q test was conducted based on the AUC values and variability calculated for each study. The test returned a result of 10.70 with a *p*-value of 0.3816, suggesting that the variation across the studies was not significantly greater than expected by chance. This indicates that the observed heterogeneity is likely due to random variation rather than real differences between the studies. Furthermore, the I^2^ statistic returned a value of 6.52%, suggesting that there was low heterogeneity among the studies, which is relatively consistent with their findings. These results were assessed and confirmed using Tau-squared (τ^2^) with a very small value of 0.000042, indicating minimal variability and the low heterogeneity. Because of the similarity between the studies and a low heterogeneity, we decided to use a fixed effects model for the meta-analysis.

However, it is important to note that these traditional heterogeneity assessments may not fully capture all aspects of variability across studies, particularly when threshold effects are present. To overcome the assumption made by using the fixed effects mode that all studies estimate the same underlying effect size and do not account for variability between studies, we used the HSROC model. Therefore, all possible variations in the thresholds that may be considered in studies for a positive diagnostic result were assessed using this model. This aspect is essential, especially when it is well known that the limitations of traditional heterogeneity measures such as Cochrane’s Q or I^2^, may not fully capture residual heterogeneity. Based on the results of these methods, such as a sensitivity of 0.942 and a specificity of 0.920 obtained from the HSROC model as well as the SROC plot, we confirmed the high diagnostic accuracy of copeptin in diagnosing polyuria-polydipsia syndrome. Through the HSROC model, we reinforced the findings of the traditional metrics.

#### 3.6.3. Publication Bias Assessment

**Figure 9:**
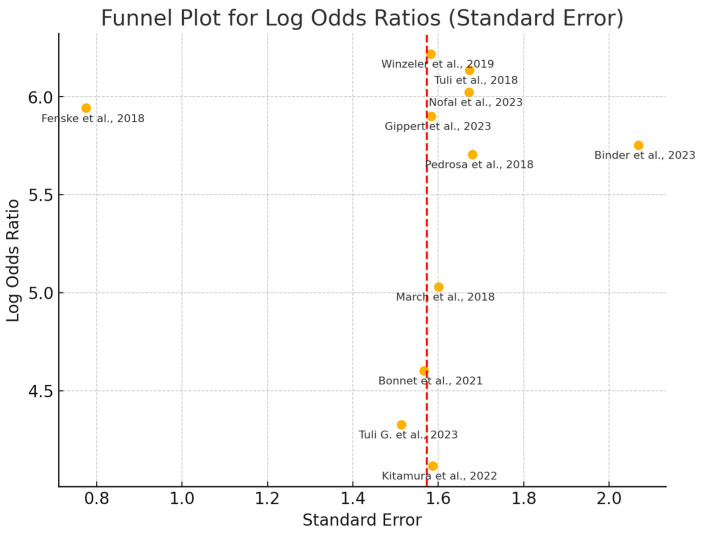
Funnel plot publication bias—it presents the Log Odds Ratios (LOR) of the included studies along with their respective confidence intervals (CIs) plotted against the standard errors (SEs). The vertical red line represents the mean LOR, while the horizontal spread of points indicates the variability in effect sizes. (Fenske et al., 2018 [10], Kitamura et al., 2022 [16], Tuli et al., 2023 [17], and Winzeler et al., 2019 [18], Binder et al., 2023 [19], Pedrosa et al., 2018 [20], Tuli et al., 2018 [21], March et al., 2018 [22], Nofal et al., 2023 [23], Gippert et al., 2023 [24], Bonnet et al. [25]).

We constructed a funnel plot (Figure 9) of the Log Odds Ratios against their standard errors to evaluate the potential for publication bias and performed Egger’s regression test. The funnel plot showed some asymmetry, which was due to the smaller studies, so there was probably some publication bias. However, Egger’s test did not show any statistical evidence of this bias (*p* = 0.716). In light of these findings, it is believed that even though there are visual cues for publication bias, the practical influence on inclusively pooled diagnostic accuracy might be low. However, this possible bias must be considered when interpreting the findings, particularly for smaller studies.

#### 3.6.4. Sensitivity Analysis Based on Risk of Bias

This analysis was performed excluding studies with a moderate risk of bias in at least two of the four domains assessed using the QUADAS-2 tool. Kitamura et al., 2022 [16], Pedrosa et al., 2018 [20], March et al., 2018 [22], and Bonnet et al., 2021 [25] had a moderate risk of either population selection or reference standard. Subsequently, the pooled sensitivity was slightly lower (0.976). However, the confidence interval between 0.9277 and 1.0250 remains very similar. Thus, the slight decrease in the overall pooled sensitivity reflects that deleting these studies with bias did not appreciably alter the overall sensitivity of copeptin in identifying true cases. After deleting studies with moderate bias, the pooled specificity increased slightly to 0.9530 with a confidence level interval of 0.9242 to 0.9818. This means that the accuracy of the diagnostic test in correctly identifying true negatives can be said to have been insufficiently improved following the elimination of biased studies. The new pooled AUC value was 0.974, with similar CIs of 0.9522 and 0.9955, respectively. The very slight increase in AUC indicates that after the exclusion of biased studies, the overall diagnostic ability did not substantially change. These results were compared with the overall analysis, which included all studies. Sensitivity analysis showed that the results were consistent and therefore proved that the inclusion of such studies in the meta-analysis did not influence the conclusion.

#### 3.6.5. Sensitivity Analysis after Excluding Adult-Inclusive Studies

Although our review and meta-analysis primarily focused on pediatric patients (children, infants and adolescents), three studies included in the meta-analysis involved both pediatric and adult patients (Fenske et al., 2018 [10], Pedrosa et al., 2018 [20], and Al Nofal et al., 2024 [23]). After screening many potential studies, only 11 were found to be eligible based on our inclusion and exclusion criteria. The main reason for including these mixed-population studies was due to the limited number of publications that focused exclusively on pediatric patients and the diagnostic utility of copeptin in polyuria-polydipsia syndrome (PPS). Although these studies included adults, they were highly relevant to pediatric populations as they examined diagnostic methods applicable to both adults and children. Therefore, their inclusion add value to our meta-analysis by providing data on diagnostic methods that are also useful for children.

To address the potential impact of including the studies conducted by Fenske et al., 2018 [10], Pedrosa et al., 2018 [20], and Al Nofal et al., 2024 [23], a sensitivity analysis was performed. After exclusion, the overall sensitivity across the remaining studies remained very high (1.0). The specificity ranged from 0.857 to 1.0, with a slightly lower pooled mean of 0.926 compared to the original dataset. The AUC values remained consistently high, with a pooled mean of 0.963 and maximum of 1.0, as reported by Binder et al. (2023) [19].

The sensitivity analysis, which excluded studies involving adult patients, confirmed that the overall diagnostic accuracy of copeptin in diagnosing pediatric PPS remains unaffected. These results suggest that inclusion of adult-inclusive studies did not introduce bias into our overall findings.

### 3.7. Sensitivity of Sub-Group Analysis for Main Diagnostic Methods

In the sensitivity analysis, we aimed to evaluate the consistency of diagnostic accuracy metrics across the different diagnostic methods used in the reviewed studies. In this way, we assessed copeptin performance as a diagnostic test among all the main diagnostic methods used for pediatric patients with polyuria-polydipsia syndrome. Specifically, we analyzed the Sensitivity, Specificity, Positive Predictive Value (PPV), Negative Predictive Value (NPV), Positive Likelihood Ratio (LR+), Negative Likelihood Ratio (LR-), and Area Under the Curve (AUC) values for baseline copeptin levels, copeptin after stimulation (hypertonic saline and arginine), and the water deprivation test.

In Group 1, we included all studies that assessed baseline copeptin levels such as Fenske et al., 2018 [10] and Pedrosa et al., 2018 [20]. For Group 2, we aimed to analyze copeptin after the hypertonic saline infusion test, and the relevant studies were Kitamura et al., 2022 [16], Tuli et al., 2023 [17], March et al., 2024 [22] and Gippert et al., 2023 [24]. In Group 3, we included the studies conducted by Winzeler et al., 2019 [18], Binder et al., 2023 [19], and Tuli et al., 2018 [21], as all these studies assess copeptin measurements after arginine stimulation. And in the final group, Group 4, we addressed the water deprivation test including the studies conducted by Bonnet et al., 2021 [25] and Nofal et al., 2023 [23].

The Kruskal–Wallis test results indicated a sensitivity of H (3) = 4.5, a *p*-value of approximately 0.21, a specificity of H (3) = 8.3 with a *p*-value of 0.40, and the results for AUG were H (3) = 5.9 with a *p*-value of 0.12. All resulting *p*-values were greater than the common significance level of 0.05, indicating that there were no statistically significant differences between the diagnostic methods for any of the metrics evaluated. This suggests that the diagnostic methods are generally consistent in their performance across studies.

Furthermore, to address the practical applications of these methods in clinical settings where prevalence may vary, we also assessed PPV and NPV along with LR+ and LR- which are valuable tools for clinicians to make decisions regarding different diagnostic methods.

Similarly, we applied the Kruskal–Wallis test, which yielded the following results: for PPV, we obtained H (3) = 4.5 with a *p*-value of 0.021; for NPV, the result was H (3) = 6.26 with a *p*-value of 0.1; for LR+, it was H (3) = 5.9 with a *p*-value of 0.11 and for LR-, the test could not be performed accurately because of the presence of perfect test values. Based on these results, there were no statistically significant differences in PPV, NPV, and LR+ across the different diagnostic methods. This suggest that the diagnostic accuracy metrics were consistent in all evaluated studies.

In this subgroup analysis, we also wanted to draw attention to the subtle differences that may not have been captured by statistical tests alone. In consequence, especially due to the fact that we used a small samples size for the Kruskal–Wallis test, we assure further assessment of the potential differences in diverse diagnostic methods using visual representations with boxplots of each diagnostic metrics.

**Figure 10:**
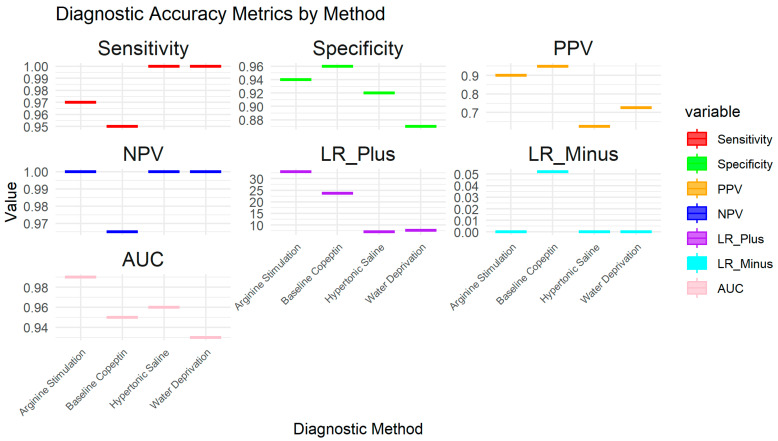
Diagnostic accuracy metrics by method—The boxplots illustrate the distribution of Sensitivity, Specificity, Positive Predictive Value (PPV), Negative Predictive Value (NPV), Positive Likelihood Ratio (LR+), Negative Likelihood Ratio (LR−), and Area Under the Curve (AUC) across the different diagnostic methods assessed in the included studies.

Visual inspection of the boxplots (Figure 10) provides additional insights. While the statistical test did not reveal significant differences, the boxplots demonstrated some variability in certain metrics, particularly PPV and specificity. These variations, although not statistically significant, may have clinical relevance, especially in scenarios where slight differences in diagnostic performance could impact patient outcomes.

**Sensitivity** shows mean values ranging from 0.95 to 1.00 across all diagnostic methods, indicating that these tests are very good at identifying patients who actually have the disease (true positives). This aspect is critical in a clinical setting to ensure that cases are not missed, and an accurate diagnosis is made.

**Specificity** shows at times some variability, with certain methods like **Baseline Copeptin Levels** and **Water Deprivation Test** displaying slightly lower medians (e.g., 0.87 to 0.96). While these methods are still very useful, the variability suggests that they may not be as effective at correctly identifying true negatives compared to the other methods. This could lead to an increase in the number of false positives in certain clinical scenarios.

**PPV** varies more widely across all diagnostic methods, particularly for the **Hypertonic Saline Infusion** and **Water Deprivation Test,** with median values ranging from 0.429 to 1.000. Therefore, these diagnostic tests may be more susceptible to the underlying prevalence of the disease in the study populations. Alternatively, the variability could be due to differences in how these tests were conducted, especially in young children. When the PPV is lower, there is a higher chance that a positive test result might be a false positive. For all diagnostic methods, the **NPV** remained very high, with median values approaching 1.00. This means that all tests are very good at ruling out the disease when the test results are negative. Such consistency across methods further underscores the reliability of these tests for identifying true negatives, even in a clinical setting.

**LR+** shows some variability, particularly with **Hypertonic Saline** and **Water Deprivation Test** methods with wider ranges (LR+ from 7.00 to 25.00). This variability suggests differences in the extent to which these methods increase the odds of disease presence when positive. **LR−** values are generally low across all methods (ranging from 0.000 to 0.052), which is desirable because lower values indicate that a negative test result effectively reduces the likelihood of disease. Although these methods are generally effective, their variability suggests that they may be more influenced by factors such as patient selection or test administration.

For all diagnostic methods, the **AUC** values were very high, with medians close to 1.00. This may indicate excellent overall average diagnostic accuracy across diagnostic methods and that all the tests compared in this study are reliable tools to diagnose polyuria-polydipsia syndrome and its related conditions in a pediatric population. A very small variability could reflect differences in the robustness of the tests under different conditions.

The observed variations underscore the importance of considering both the statistical significance and practical relevance in clinical settings. Although the diagnostic methods appear consistent overall, the visual analysis highlights areas where specific methods may perform differently and evaluate subtle variances that statistics may not cover. This can guide future research and clinical practice. For instance, despite the general statistical analysis where all methods are effective, Baseline Copeptin Levels and Copeptin After Arginine Stimulation showed the most consistent results across all metrics, even in the visual analysis. This could make them more reliable choices in clinical settings where consistency is critical.

## 4. Discussion

### 4.1. Principal Findings

Although diagnosis of polyuria-polydipsia syndrome has been widely studied in adult patients, we faced a limited number of studies addressing this pathology in children and adolescents. Even if we thoroughly searched several databases, after rigorous assessment of the potentially suitable studies, we had to exclude many studies because these articles did not focus on pediatric patients or were analyzing tests that did not meet our interest and objectives. Therefore, in our systematic review and meta-analysis, we rigorously evaluated the diagnostic accuracy of copeptin measurements, both baseline and post-stimulation, as an alternative to the traditional water deprivation test (WDT) in pediatric patients with polyuria-polydipsia syndrome (PPS).

By analyzing the results of the 11 studies that met the PIT inclusion criteria, we consistently demonstrated that copeptin is a highly effective diagnostic biomarker, with a pooled sensitivity of 0.98 (95% CI: 0.936–1.025), a pooled specificity of 0.947 (95% CI: 0.920–0.973), and an AUC of 0.972 (95% CI: 0.952–0.992), indicating excellent diagnostic accuracy.

The heterogeneity assessment proved to be low (Cochran’s Q = 10.70, *p* = 0.382; I^2^ = 6.52%; τ^2^ = 0.000042) and the meta-analysis was based on a fixed effects model. However, we further assessed all the potential residual variances due to different thresholds in diagnostic tests by using the HSROC model to reinforce our findings.

The results obtained through this more complex statistical model showed a logit-transformed sensitivity of 2.7873 and a logit-transformed false positive rate of −2.4335, corresponding to an overall sensitivity of 0.942 and a specificity of 0.92. The data helped us to obtain a more complex picture of the diagnostic accuracy of copeptin. The HSROC model helps to deal with interstudy variability and the differences in how studies are conducted. This allowed for a more accurate reflection of the diagnostic test’s performance across different practical contexts, making these results more reliable for clinical decisions. Both statistical approaches confirmed that copeptin is a valuable diagnostic test for accurately establishing the etiological diagnosis of polyuria-polydipsia syndrome in pediatric patients.

Due to the fact that we recorded high values for sensitivity and specificity, as well as because of a limited sample size of studies included, assessing bias was an essential target in our review and meta-analysis in order to search for all potential factors that could interfere with the interpretation of these results in terms of diagnostic accuracy and efficacy. Publication bias was assessed using a funnel plot for visual assessment (LOR of the included studies were plotted against their SE), which demonstrated slight asymmetry especially due to some studies (Fenske et al., 2018 [10], Binder et al., 2023 [19]) that had differences in study design or populations. Egger’s test (*p* = 0.716) was performed and both methods confirmed that there was no publication bias in the selected studies. To ensure that the selected studies were classified as having moderate bias in two of the four criteria after QUADAS-2 assessment, we performed a sensitivity analysis to evaluate their impact on the total sample of studies. Studies conducted by Kitamura et al., 2022 [16]; Pedrosa et al., 2018 [20]; March et al., 2018 [22]; and Bonnet et al., 2021 [25] were excluded, and the sensitivity analysis results were consistent (sensitivity = 0.976, 95% CI: 0.928–1.025; specificity = 0.953, 95% CI: 0.924–0.982; AUC = 0.974, 95% CI: 0.952–0.996), indicating that the inclusion of such studies in the meta-analysis did not influence outcomes. In addition, due to the limited number of pediatric-only studies on the diagnostic utility of copeptin in PPS, three studies with both children and adult patients were included. Sensitivity analysis excluding adult-inclusive studies revealed no significant changes in diagnostic accuracy (pooled mean sensitivity—1.00; pooled specificity—0.925; and pooled AUC—0.963). This strengthens the conclusion that copeptin is a reliable biomarker for the diagnosis of pediatric PPS.

### 4.2. Comparison with Previous Studies

The diagnosis of PPS in the pediatric population represents a real challenge for clinicians especially in young children where standard WDT is difficult to conduct following all protocol steps. As a result, the findings in this review and meta-analysis align with those of previous studies in which copeptin testing is a useful alternative diagnostic tool in the pediatric population. It has been reported that the copeptin levels are not corelated with age, and some thresholds in children are similar to those established in the adult population, but these aspects need to be further proven by prospective studies in the pediatric population [25]. In fact, differential diagnosis and establishing the form of polydipsia-polyuria syndrome is a more complex medical-thinking process. A principal diagnostic of CDI in children by Bonnet et al., 2021 [25] and Winzeler et al., 2019 [18] reported the use of a copeptin threshold of 3.53 pmol/L having a sensitivity of 100% and a specificity of 87.4%, and copeptin dosing after arginine stimulation test with a cutoff of 3.8 pmol/L with a sensitivity of 93% and a specificity of 92%. These findings are similar to those of the present study. Statistical sensitivity analysis and visual assessment of boxplots for each diagnostic method suggested higher accuracy for the arginine stimulated copeptin test, achieving better diagnostic metrics in comparison to standard WDT. NDI is reported to have a lower frequency in the pediatric population (estimated prevalence in males 8.8: 1.000.000) [26]. To differentiate this pathological entity, Tuli et al., 2018 [21] concluded that copeptin values >20 ymol/L after WDT are pathognomonic for NDI [21]. Primary polydipsia remains, as reported in the scientific literature, an important differential diagnosis for children with PPS. In this case, the copeptin level measured after hypertonic saline infusion proved to be more accurately and more reliable to differentiate primary polydipsia from central diabetes insipidus (Fenske et al.—Copeptin cutoff > 4.9 pmol/L: sensitivity = 93.2% [95% CI: 83.5–98.1], specificity = 100% [95% CI: 95.5–100.0], AUC = 0.97 [95% CI: 0.93–1.00]) than the water-deprivation test with or without copeptin measurement.

When discussing the differential diagnosis of these cases, we should consider other rare causes of PPS, such as systemic autoimmune inflammatory diseases [27,28], Langerhans histiocytosis [29], cerebral tumors [30,31], head trauma associated with the syndrome of inappropriate secretion of antidiuretic hormone (SIADH) [32], or other renal impairments [33]. Additionally, severe cases of sepsis or multisystemic inflammatory syndrome in children (MIS-C) are associated with abnormal ADH secretion [34,35,36]. The complex pathophysiological processes that involve ADH secretion from the neurohypophysis have a direct impact on water balance and hydroelectrolitic impairment. Copeptin dosing has been reported as a reliable biomarker for these diagnosis of pediatric septic shock (sensitivity = 94%, ROC = 0.960—Saleh et al., 2023 [35]). This is because of the well-described biphasic ADH secretion in the early stages of septic shocks, especially in relation to hyponatremia, and hemodynamic instability [35,37].

In these cases, copeptin has been reported to have important diagnostic implications, which account for our results derived from the SROC curve (Figure 8) generated by the HSROC model. The SROC curve shows the plotted sensitivity versus 1—Specificity, representing the copeptin test’s ability to discriminate between cases and non-cases across different thresholds and clinical scenarios, such as this differential diagnosis associated with ADH secretion abnormalities.

This review further corroborates the utility of copeptin across diverse pediatric populations and clinical settings, extending its applicability beyond what has been reported in earlier studies. Furthermore, we assessed all the main diagnostic methods to provide a new perspective regarding the clinical approach and selection of these tests.

### 4.3. Clinical Implications

One of the objectives of this review and meta-analysis was to assess the efficiency and diagnostic accuracy of the primary diagnostic methods for PPS in children. Therefore, we conducted a sensitivity subgroup analysis for baseline copeptin levels, copeptin after stimulation with hypertonic saline solution or arginine stimulation, and the water deprivation test. The statistical results regarding the diagnostic metrics for each method have a direct impact and relevance in clinical practice. The Kruskal–Wallis test, a non-parametric test, was used to assess the main metrics for diagnostic accuracy consistency because the data were not normally distributed, as shown by the Shapiro–Wilk test. The Kruskal–Wallis test showed (Sensitivity: H(3) = 4.5, *p* = 0.21; Specificity: H(3) = 8.3, *p* = 0.40; AUC: H(3) = 5.9, *p* = 0.12) no statistically significant difference for any of the diagnostic methods evaluated in terms of general performance. To further investigate the practical implications of these methods in clinical settings where prevalence may vary in pediatric patients, we also conducted the Kruskal–Wallis test for PPV and NPV, along with LR+ and LR− (PPV: H(3) = 4.5, *p* = 0.021; NPV: H(3) = 6.26, *p* = 0.10; LR+: H(3) = 5.9, *p* = 0.11; LR− irrelevant), again proving that there was no statistically significant difference.

Considering that clinical experience in conducting these diagnostic tests may vary on different aspects and situations, we aimed to address the practical applicability of all these methods as thoroughly as possible. Consequently, we used combined statistical and visual (boxplot) analysis to investigate all differences between these diagnostic tests. The high sensitivity of all the methods indicates that they are reliable tools for detecting PPS. Therefore, they are particularly useful in clinical situations where accurate diagnosis is critical. However, the slight variability in specificity, especially at the level of baseline copeptin levels and the water deprivation test, suggests that clinicians should be cautious and suspicious of false positives, especially in ruling out the condition. The range for the PPV, especially for the hypertonic saline infusion and water deprivation test, suggests that these methods might be more prone to identify false positives in populations with a low prevalence of PPS. However, all methods have a high NPV, thus making them reliable for ruling out PPS, which is important for clinical decision-making. The variability in LR+ indicates that while these tests generally increase the likelihood of diagnosing PPS when positive, the degree to which they do so may vary depending on the test used and clinical context. The low values of LR− for all these methods confirm their usefulness for unequivocal exclusion of PPS when the test is negative. The high AUC values and good reliability of all these methods indicate that they are valuable diagnostic tools for PPS. However, the observed variability in some metrics, although not statistically significant, highlights the importance of considering both the statistical outcomes and practical clinical relevance when selecting a diagnostic method.

The clinical implications of our findings are significant. The high diagnostic accuracy of copeptin, particularly post-stimulation with arginine, suggests that it can effectively replace WDT in most young children, reduce patient discomfort, and improve diagnostic workflows in pediatric patients. This is especially relevant in settings where the WDT is impractical or difficult to realize due to clinical impediments to follow the exact protocol, especially in young patients who are suffering from different types of PPS and need to be accurately diagnosed.

### 4.4. Limitations

Despite these consistent findings, this study had several limitations. The review and meta-analysis were based on a limited number of studies that met all the inclusion criteria to select the most reliable ones for a solid analysis. Although a comprehensive statistical evaluation of these studies indicated a low risk of bias, some concerns and limitations might be considered regarding the differences in patient populations, study design, and copeptin measurement protocols. One potential limitation is the inclusion of studies with mixed pediatric and adult populations. However, we conducted a sensitivity analysis excluding these studies which showed no significant impact on diagnostic accuracy.

Another aspect that could be considered is the retrospective nature of some of the included studies which could have induced some degree of bias, particularly in patients’ selection and reference standard application.

Finally, although copeptin showed consistency in diagnostic accuracy, the different diagnostic test thresholds emphasize the utility of introducing a standardized testing protocol.

## 5. Conclusions and Future Perspectives

Our review asserts that copeptin dosing, especially after stimulation tests, has significant clinical value, representing a reliable, more comfortable and less stressful diagnostic tool for PPS in pediatric patients. Sensitivity analysis indicated that baseline copeptin levels and copeptin levels after arginine stimulation provided the most consistent diagnostic performance. These methods are preferred in clinical situations where accuracy is crucial. The variability observed in hypertonic saline and water deprivation tests suggests that further research should be conducted to optimize these approaches, especially in young children. Furthermore, future studies are needed to improve these methods for broader clinical use, refine copeptin thresholds, and develop a diagnostic protocol for children with PPS.

## Figures and Tables

**Table 1:** Key selected studies.

Study	Citation	Participants	Clinical Setting	Study Design	Target Condition	Index Test	Reference Standard	Sample Size	Funding Sources
1	Fenske et al., 2018 [10]	156 patients aged 16 years or older	11 tertiary medical centers in Switzerland and Germany	Prospective multicenter study	Hypotonic polyuria	Copeptin measurement after water deprivation and hypertonic saline tests	Final reference diagnosis by expert consensus. Clinical diagnosis based on established criteria for central diabetes insipidus (CDI) and primary polydipsia (PP)	156 participants	Supported by Swiss National Foundation, University Hospital Basel, Federal Ministry of Education and Research, Germany, Deutsche Forschungsgemeinschaft, Thermo Fisher Scientific
2	Kitamura M, et al., 2022 [16]	27 patients with polyuria and/or polydipsia (5 with central diabetes insipidus, 5 with primary polydipsia, 1 with nocturnal enuresis, and 16 with type 1 diabetes mellitus (excluded due to hyperglycemia)	Department of Pediatrics and Child Health, Kurume University School of Medicine, Japan	Retrospective chart review	Central diabetes insipidus (CDI), primary polydipsia (PP), nocturnal enuresis	Hypertonic saline test, urine gravity in the morning	Copeptin levels, urine volume over 24 h, urinary osmolality, plasma osmolality	27 participants	Not specified
3	Tuli, G., Munarin, J., De Sanctis, L. (2023) [17]	Children with PPS including 7 with primary polyuria (PP), 6 with central diabetes insipidus (CDI), and 50 control subjects	Department of Pediatric Endocrinology, Regina Margherita Children Hospital	Prospective study	Central diabetes insipidus (CDI), primary polyuria (PP)	Arginine-stimulated copeptin test	Water deprivation test (WDT)	63 participants (13 patients with PPS, 50 controls)	Not specified
4	Winzeler B., et al., 2019 [18]	Development cohort: 52 patients (12 with complete DI, 9 with partial DI, 31 with PP), 20 healthy adults, and 42 child controls; validation cohort: 46 patients (12 with complete DI, 7 with partial DI, 27 with PP) and 30 healthy adult controls	Multi-center study involving University Hospital Basel and five other centers in Switzerland and Germany	Prospective diagnostic study	Complete and partial diabetes insipidus, primary polydipsia	Arginine-stimulated copeptin measurements	Diagnostic accuracy of copeptin levels	Development cohort: 114 participants; validation cohort: 76 participants (after exclusions)	Swiss National Science Foundation and University Hospital Basel
5	Binder, G., Weber, K., Peter, A., and Schweizer, R. (2023) [19]	72 children and adolescents tested for growth hormone deficiency, including 4 with confirmed central diabetes insipidus (CDI)	University Children’s Hospital Tübingen, Germany	Monocentric retrospective analysis	Central diabetes insipidus (CDI)	Arginine-stimulated copeptin test	Water deprivation test, serum osmolality arginine-stimulation test and clinical evaluation	72 participants (68 non-CDI, 4 with CDI)	Not specified
6	Pedrosa et al., 2018 [20]	52 patients aged 12–77 years	Hermes Pardini Endocrine Testing Center, Brazil	Retrospective analysis	Polyuria-polydipsia syndrome (PPS)	Hypertonic saline infusion followed by copeptin measurement	Plasma AVP levels	52 participants	Supported by FAPEMIG and CNPq, JBD received a PhD grant from CAPES
7	Tuli, G., Munarin, J., De Sanctis, L., 2018 [21]	80 children (53 control subjects, 12 hypopituitary children, and 15 children with PPS)	Pediatric Endocrinology Department	Prospective observational study	Nephrogenic diabetes insipidus (NDI), complete central diabetes insipidus (CDI), and primary polydipsia (PP)	Copeptin levels after hypertonic saline infusion test and water deprivation test	Plasma arginine vasopressin (AVP) analysis	80 participants	Not specified
8	March, C. A., Sastry, S., McPhaul, M. J., Wheeler, S. E., and Garibaldi, L. (2024) [22]	47 healthy short children (controls), 10 children with primary polydipsia, and 10 children with AVP deficiency	Pediatric endocrinology settings	Prospective interventional study	Polyuria-polydipsia syndrome (PPS), arginine vasopressin (AVP) deficiency, primary polydipsia	Arginine + LevoDopa/Carbidopa stimulation test (ALD-ST) for copeptin measurement	Copeptin levels measured at baseline and after stimulation	67 participants (47 controls, 10 with primary polydipsia, 10 with AVP deficiency)	Not specified
9	Al Nofal, A., et al. (2024) [23]	29 critically ill patients, including 6 infants, with hyper- or hypo-natremia	Single-center study conducted in a hospital setting	Retrospective case series	Central diabetes insipidus (CDI), syndrome of inappropriate antidiuresis (SIAD)	Copeptin levels after hypertonic saline test	Diagnostic thresholds for CDI and SIAD	29 patients (38% post-neurosurgical procedures)	Not specified
10	Gippert, S., Brune, M., Dirksen, R. L., Choukair, D., Bettendorf, M. (2024) [24]	69 patients (32 with central diabetes insipidus [CDI], 32 matched controls, and 5 with primary polydipsia [PP])	Single-center study in a pediatric endocrinology department	Retrospective study	Central diabetes insipidus (CDI), primary polydipsia (PP)	Copeptin measurement following water deprivation test	Comprehensive clinical and diagnostic characteristics, water deprivation test, and hypertonic saline infusion	69 participants	Not specified
11	Bonnet, L., et al., 2024 [25]	353 children aged 2 months to 18 years	Pediatric endocrinology and nephrology departments in France	Single-center retrospective descriptive study	Central diabetes insipidus (CDI), primary polydipsia (PP), and other related conditions	Copeptin levels measured after arginine stimulation	Comparison with traditional diagnostic criteria for CDI and PP	353 participants, with 16 diagnosed with CDI and 18 with PP	Funded by the University of Pittsburgh Clinical and Translational Science Institute Clinical and Translational Science Scholars Program (NIH/NCATS 1 KL2 TR001856)

**Table 2:** Results of individual studies.

Study	TP *	FN *	TN *	FP *	Sensitivity (95% CI)	Specificity (95% CI)	PPV (95% CI)	NPV (95% CI)	LR+ (95% CI)	LR− (95% CI)	AUC (95% CI)
1 Fenske et al., 2018 [10]	56	3	82	3	0.95 (95% CI: 85.15–99.36%)	0.96 (96% CI: 89.73–98.89%)	0.949 CI: (0.902–0.996)	0.965 CI: (0.926–1.000)	23.75 CI: (6.91–81.57)	0.052 CI: (0.011–0.241)	0.95 CI: (0.88–0.98)
2 Kitamura et al., 2022 [16]	5	0	19	3	1 (100% CI: 56.56–100%)	0.857 (85.7% CI: 64.54–95.85%)	0.625 CI: (0.396–0.854)	1.000 CI: (0.829–1.000)	7.00 CI: (2.55–19.24)	0.000 CI: (0.00–0.00)	0.93 CI: (0.85–0.99)
3 Tuli G. et al., 2023 [17]	6	0	49	8	1 (100% CI: 61.51–100%)	0.857 (85.7% CI: 72.25–93.37%)	0.429 CI: (0.237–0.621)	1.000 CI: (0.928–1.000)	7.00 CI: (2.55–19.24)	0.000 CI: (0.00–0.00)	0.93 CI: (0.83–0.98)
4 Winzeler et al., 2019 [18]	11	0	54	2	1 (100% CI: 71.51–100%)	0.96 (96% CI: 84.98–99.46%)	0.846 CI: (0.709–0.983)	1.000 CI: (0.932–1.000)	25.00 CI: (6.32–98.89)	0.000 CI: (0.00–0.00)	0.98 CI: (0.92–1.00)
5 Binder et al., 2023 [19]	4	0	17	0	1 (100% CI: 56.56–100%)	1 (100% CI: 76.03–100%)	1.000 CI: (0.783–1.000)	1.000 CI: (0.803–1.000)	N/A (perfect tets)	N/A (perfect tets)	1.00 (perfect test) CI: (0.90–1.00)
6 Pedrosa et al., 2018 [20]	8	0	26	1	1 (100% CI: 56.56–1000%)	0.96 (96% CI: 77.97–99.57%)	0.889 CI: (0.679–1.000)	1.000 CI: (0.867–1.000)	25.00 CI: (3.66–171.14)	0.000 CI: (0.00–0.00)	0.98 CI: (0.92–1.00)
7 Tuli et al., 2018 [21]	9	0	36	1	1 (100% CI: 61.51–100%)	0.97 (97% CI: 83.34–99.63%)	0.900 CI: (0.742–1.000)	1.000 CI: (0.889–1.000)	33.00 CI: (4.72–230.84)	0.000 CI: (0.00–0.00)	0.99 CI: (0.92–1.00)
8 March et al., 2018 [22]	8	0	22	2	1 (100% CI: 61.51–100%)	0.92 (92% CI: 69.99–98.98%)	0.800 CI: (0.543–1.000)	1.000 CI: (0.824–1.000)	12.50 CI: (3.46–45.13)	0.000 CI: (0.00–0.00)	0.96 CI: (0.88–0.99)
9 Nofal et al., 2023 [23]	10	0	29	1	1 (100% CI: 69.15–100%)	0.95 (95% CI: 80.53–99.29%)	0.909 CI: (0.725–1.000)	1.000 CI: (0.868–1.000)	20.00 CI: (4.65–86.08)	0.000 CI: (0.00–0.00)	0.98 CI: (0.90–0.99)
10 Gippert et al., 2023 [24]	12	0	36	2	1 (100% CI: 73.54–100%)	0.94 (94% CI: 80.09–98.40%)	0.857 CI: (0.689–1.000)	1.000 CI: (0.917–1.000)	16.67 CI: (5.15–53.89)	0.000 CI: (0.00–0.00)	0.97 CI: (0.89–0.99)
11 Bonnet et al., 2021 [25]	8	0	20	3	1 (100% CI: 56.56–100%)	0.87 (87% CI: 66.89–95.45%)	0.727 CI: (0.476–0.978)	1.000 CI: (0.823–1.000)	7.69 CI: (2.86–20.64)	0.000 CI: (0.00–0.00)	0.93 CI: (0.82–0.97)

* TP—true positive; FN—false negative; TN—true negative; FP—false positive.

**Table 3:** Variances and weights.

Study	Sensitivity	Specificity	AUC (95% CI)	Sensitivity Variance	Specificity Variance	AUC Variance	Weight (Sensitivity)	Weight (Specificity)	Weight (AUC)
1 Fenske et al., 2018 [10]	0.95	0.96	0.95	0.0013	0.000548	0.000423	769.23	1824.82	2364.07
2 Kitamura et al., 2022 [16]	1.00	0.857	0.93	0.0123	0.0064	0.006735	81.30	156.25	148.48
3 Tuli G. et al., 2023 [17]	1.00	0.857	0.93	0.0097	0.0029	0.005389	103.09	344.83	185.56
4 Winzeler et al., 2019 [18]	1.00	0.96	0.98	0.0053	0.0014	0.000904	188.68	714.29	1106.19
5 Binder et al., 2023 [19]	1.00	1.00	1.00	0.0123	0.0037	0.001	81.30	270.27	1000.0
6 Pedrosa et al., 2018 [20]	1.00	0.96	0.98	0.0123	0.0030	0.001271	81.30	333.33	786.78
7 Tuli et al., 2018 [21]	1.00	0.97	0.99	0.0097	0.0017	0.000565	103.09	588.24	1769.91
8 March et al., 2018 [22]	1.00	0.92	0.96	0.0097	0.0054	0.002507	103.09	185.19	398.88
9 Nofal et al., 2023 [23]	1.00	0.95	0.98	0.0062	0.0023	0.001015	161.29	434.78	985.22
10 Gippert et al., 2023 [24]	1.00	0.94	0.97	0.0046	0.0022	0.001247	217.39	454.55	801.92
11 Bonnet et al., 2021 [25]	1.00	0.87	0.93	0.0123	0.0053	0.004267	81.30	188.68	234.36

## Appendix A

The table below outlines the search strategies used for each database in this systematic review. It includes search strings and the limitations applied to ensure transparency and reproducibility.

**Table A1:** Search strategy.

Database	Search String	Limits
PubMed	((“Polyuria”[Mesh] OR “Polydipsia”[Mesh] OR “Central Diabetes Insipidus”[Mesh] OR “Primary Polydipsia”[Mesh] OR “Nephrogenic Diabetes Insipidus”[Mesh]) AND (“Arginine Vasopressin”[Mesh] OR “C-Terminal Provasopressin”[Mesh] OR “Water Deprivation Test”[Mesh] OR “Desmopressin”[Mesh] OR “Copeptin”[Mesh] OR “Baseline Copeptin” OR “Copeptin Stimulation” OR “Copeptin Test” OR “Saline Infusion Test” OR “Arginine Stimulation”) AND (“Child”[Mesh] OR “Adolescent”[Mesh]) AND (“Diagnostic Accuracy” OR “Sensitivity” OR “Specificity” OR “ROC” OR “AUC” OR “Predictive Value”))	Publication date from 2018 to 2024, Humans, Children and Adolescents
Cochrane Library	((Polyuria OR Polydipsia OR “Central Diabetes Insipidus” OR “Primary Polydipsia” OR “Nephrogenic Diabetes Insipidus”) AND (“Arginine Vasopressin” OR “C-Terminal Provasopressin” OR “Water Deprivation Test” OR “Desmopressin” OR “Copeptin” OR “Baseline Copeptin” OR “Copeptin Stimulation” OR “Copeptin Test” OR “Saline Infusion Test” OR “Arginine Stimulation”) AND (Child OR Adolescent) AND (“Diagnostic Accuracy” OR “Sensitivity” OR “Specificity” OR “ROC” OR “AUC” OR “Predictive Value”))	Publication date from 2018 to 2024, Trials, Reviews
Web of Science	(TS = (Polyuria OR Polydipsia OR “Central Diabetes Insipidus” OR “Primary Polydipsia” OR “Nephrogenic Diabetes Insipidus”) AND TS = (“Arginine Vasopressin” OR “C-Terminal Provasopressin” OR “Water Deprivation Test” OR “Desmopressin” OR “Copeptin” OR “Baseline Copeptin” OR “Copeptin Stimulation” OR “Copeptin Test” OR “Saline Infusion Test” OR “Arginine Stimulation”) AND TS = (Child OR Adolescent) AND TS = (“Diagnostic Accuracy” OR “Sensitivity” OR “Specificity” OR “ROC” OR “AUC” OR “Predictive Value”))	Timespan: 2018–2024, Indexes: SCI-EXPANDED, SSCI, A&HCI, ESCI
ScienceDirect	Search String 1: (Polyuria OR Polydipsia OR “Central Diabetes Insipidus” OR “Primary Polydipsia”) AND (Copeptin OR “Arginine Vasopressin” OR “Water deprivation test”) AND (Child OR Adolescent)Search String 2: (“Water Deprivation Test” OR Desmopressin OR “Copeptin Stimulation” OR “Saline Infusion Test”) AND (“Central Diabetes Insipidus” OR “Nephrogenic Diabetes Insipidus” OR “Primary Polydipsia”) AND (Child OR Adolescent)	Date: 2018–2024, Article type: Research Articles
Scopus	TITLE-ABS-KEY(Polyuria OR Polydipsia OR “Central Diabetes Insipidus” OR “Primary Polydipsia” OR “Nephrogenic Diabetes Insipidus”) AND TITLE-ABS-KEY(“Arginine Vasopressin” OR “C-Terminal Provasopressin” OR “Water Deprivation Test” OR “Desmopressin” OR “Copeptin” OR “Baseline Copeptin” OR “Copeptin Stimulation” OR “Copeptin Test” OR “Saline Infusion Test” OR “Arginine Stimulation”) AND TITLE-ABS-KEY(Child OR Adolescent) AND TITLE-ABS-KEY(“Diagnostic Accuracy” OR “Sensitivity” OR “Specificity” OR “ROC” OR “AUC” OR “Predictive Value”)	Limits:Date: 2018–2024, Document type: Article; Humans; Child
Google Scholar	(Polyuria OR Polydipsia OR “Central Diabetes Insipidus” OR “ Nephrogenic Diabetes Insipidus” OR “Primary Polydipsia”) AND (Copeptin OR “Arginine Vasopressin” OR “Water deprivation test”) AND (Child OR Adolescent) AND (Diagnosis OR “Diagnostic Accuracy” OR Sensitivity OR Specificity OR “ROC Curve”) -treatment -meta-analysis -case-report	Date: 2018–2024

## Appendix B. Independent Read and Assessment of Quality Using QUADAS-2

**Independent Assessment:** In each review, the two reviewers independently assessed the risk of bias and applicability using the QUADAS-2 tool. Any disagreement was resolved through discussion or consultation with a third reviewer to reach an agreement.
**Data Extraction Forms:** The data extraction forms were piloted in a few studies to determine consistency and clarity. The extracted data included items on study design, patient demographics, details of the index test and reference standard, and measures of diagnostic accuracy. This allowed data extraction in all studies reviewed for the overview to be systematic and comprehensive.
**QUADAS-2 tool domains:** The QUADAS-2 tool was run against these studies with respect to four key domains:
**Patient Selection:** The review examined how participants were selected and the appropriateness of the selection criteria in projecting the possibility of selection bias. Common sources of bias were observed to originate from the retrospective study designs and small populations.
**Index Test:** The focus was on the diagnostic test being studied, carried out and interpreted; in particular, whether investigators applied the reference standard blind. There are various methods of copeptin measurement and stimulation protocols across studies.
**Reference Standard:** This domain examines the validity and applicability of the reference standard used to classify the subjects regarding the target condition. The reliability of standards such as WDT and plasma arginine vasopressin analysis was generally well supported, although some studies varied in their application. In some cases, the reference standard was based on expert opinion without additional objective measures that could introduce some subjectivity or was based on clinical judgment without blinding, which could also induce bias.
**Flow and Timing.** In the methodology assessment, it was examined whether investigations were carried out with appropriate timing between the index test and the reference standard. Retrospective study designs often yield variability in timing, whereas prospective designs were very rare. Timing was usually adequate across studies; however, because of the variability in timing between the index test and the reference standard in retrospective studies, the results of some of these studies may not be generalized to clinical practice.
**Quality Assessment Findings:** Results relating to the risk of bias and concerns relating to the appraisal of applicability are summarized in one structured table (Appendix C, Table A2), indicating areas of potential bias and issues with respect to generalizability as well as graphic representation using “traffic light” plots (Figure 1) of the domain-level judgements for each individual study, created using the ROBVIS tool.
**Sensitivity Analysis:** A sensitivity analysis was performed to assess the contribution of studies with medium risk of bias in the overall results. The exclusion of studies with noticeable bias did not materially alter the overall findings, thus supporting the fact that the conclusions of this review are robust despite the identified risks of bias.
**Applicability Issues:** Patient selection: The studies differed in age range, with most targeting the pediatric population. Nevertheless, some of the studies included adults and pediatric patients; therefore, generalization of the results to a pediatric population may be diminished.
**Index test conduct:** Possible differences in the performance characteristics of copeptin measurement may bear on the modes of stimulation that affect its generalizability. Thus, standardization of test procedures is suggested for better comparability in further studies.
**Reference Standard:** In general, the test protocols were consistent and reliable. The use of expert judgement without blinding is likely to influence study comparability. Future studies should strive toward greater consistency in applying reference standards to enhance the reliability of the pooled analyses.
**Flow and Timing:** While generally appropriate, the retrospective design of some studies probably introduced potential variability in the timing of index tests and reference standards, which may affect the applicability of these findings to routine clinical practice. For this reason, future studies should establish the timing in a way that delineates consistency so that the results are more reliable and applicable.

## Appendix C

**Table A2:** QUADAS-2 assessment summary.

Study	Patient Selection (Risk of Bias)	Index Test (Risk of Bias)	Reference Standard (Risk of Bias)	Flow and Timing (Risk of Bias)	Overall Summary of Bias	Observations
1. Fenske et al., 2018 [10]	Low (some concerns on applicability)	Low	Low (minor subjectivity)	Low	Low overall risk	The study involved patients from tertiary centers, which may not represent the general population.
2. Kitamura et al., 2022 [16]	Moderate (retrospective, single-center)	Low to Moderate (potential blinding issues)	Moderate (expert consensus)	Low	Moderate overall risk	Retrospective design and single-center setting increase the selection bias; possible lack of blinding.
3. Tuli et al., 2023 [17]	Low	Low	Low	Low	Low overall risk	Prospective design with a well-defined patient population, reducing bias.
4. Winzeler et al., 2019 [18]	Low (concerns about applicability to pediatric)	Low	Low to Moderate (subjectivity)	Low	Low overall risk	The study is focused on adults, limiting applicability to pediatric populations.
5. Binder et al., 2023 [19]	Low	Low	Low	Low	Low overall risk	Clear protocol and prospective design contribute to low bias across domains.
6. Pedrosa et al., 2018 [20]	Moderate (retrospective, single-center)	Low	Moderate (subjectivity)	Low	Moderate overall risk	Retrospective design and reliance on expert opinion without blinding introduce moderate bias.
7. Tuli et al., 2018 [21]	Low	Low	Moderate (subjectivity)	Low	Low overall risk	Prospective study with rigorous methods, though expert consensus introduces some subjectivity.
8. March et al., 2018 [22]	Low to Moderate (single-center setting)	Low	Moderate (subjectivity)	Low	Low to Moderate overall risk	Single-center design may affect generalizability, and expert consensus introduces subjectivity.
9. Nofal et al., 2023 [23]	Low	Low	Moderate (subjectivity)	Low	Low overall risk	Multicenter design reduces selection bias, but expert consensus may introduce some subjectivity.
10. Gippert et al., 2023 [24]	Low	Low	Moderate (subjectivity)	Low	Low overall risk	Consistent methods across centers, though reliance on expert consensus introduces moderate bias.
11. Bonnet et al., 2024 [25]	Low to Moderate (single-center setting)	Low	Moderate (subjectivity)	Low	Low to Moderate overall risk	Single-center design could limit generalizability; expert consensus introduces moderate bias.
